# Understanding midwifery workforce recruitment and retention: a realist review protocol for synthesising international evidence to inform UK workforce strategy

**DOI:** 10.1136/bmjopen-2026-124745

**Published:** 2026-10-20

**Authors:** Heidi Stelling, Megan E L Brown, Alex Boyle, Christina Higgins, Chris Dodd, Erin Todd, Eleanor Hoverd, Sophie Park, Bryan Burford, Gillian Vance

**Affiliations:** 1 School of Medicine, Newcastle University, Newcastle upon Tyne, Tyne and Wear, UK; 2 Nuffield Department of Primary Care Health Sciences, University of Oxford, Oxford, UK

**Keywords:** Midwifery, Health Workforce, Systematic Review

## Abstract

**Abstract:**

**Introduction:**

Midwives play an essential role in improving maternal and newborn outcomes, yet workforce shortages continue to challenge the sustainability of maternity services internationally and within the UK. An earlier scoping review by the authors identified multiple barriers, enablers and workforce strategies associated with recruitment and retention. However, considerable variation in findings suggested that factors influencing recruitment and retention may operate differently according to context. Existing evidence offers limited explanatory understanding regarding how, why, for whom and under what circumstances factors shaping recruitment and retention of midwives influence workforce sustainability. This protocol outlines a realist review designed to develop programme theory explaining the contextual and causal factors that may influence entry into the midwifery profession and retention of midwives.

**Methods and analysis:**

The review will commence in mid-2026 and be completed by early 2027. It will take a realist approach following Pawson’s six iterative stages of realist synthesis. Candidate theories, an initial programme theory, and the realist research question are informed by our earlier international scoping review and engagement with expert advisors across two panels comprising midwifery staff and workforce stakeholders. Searching will proceed iteratively and include database searching, citation tracking, targeted concept searching and grey literature. Evidence will be appraised according to relevance, richness and rigour and data extraction and synthesis will follow a realist logic of analysis.

**Ethics and dissemination:**

The review will involve analysis of existing published and publicly available data and therefore does not require formal ethical approval. Experts contributing to theory development will be involved as advisors rather than research participants. Findings will be disseminated through peer reviewed publication, conference presentation and stakeholder-focused outputs. The review will generate evidence-informed programme theory to support future midwifery workforce policy and practice.

**PROSPERO registration details:**

CRD420261398706.

**STRENGTHS AND LIMITATIONS OF THIS STUDY**

- Candidate theories and an initial programme theory will be developed from a preceding international scoping review and input from two expert advisory panels.
- The search will combine bibliographic database searching, citation searching, targeted concept searching and grey-literature searching, with further searches guided by emerging theory.
- Stakeholder engagement will be embedded throughout the review process supporting refinement of programme theory through perspectives from midwives, workforce stakeholders and public contributors.
- The review will be limited to English language sources, which may exclude potentially relevant evidence and explanatory insights from non-English contexts.
- As realist searching is theory-driven rather than exhaustive, some potentially relevant sources may not be identified.

## Introduction

 Midwives play a central role in improving maternal and newborn outcomes, contributing to lower medical intervention rates during labour and preventable morbidity and mortality through the provision of skilled, relationship-based care.^1–4^ International evidence demonstrates that investment in midwifery is associated with improved outcomes for women and infants, alongside wider health system benefits, including improved quality and safety of care, more efficient use of health-system resources and strengthened maternity workforce capacity.^5–7^ Despite this, midwife workforce shortages are a persistent international challenge, including within the UK.^8–10^ In England, declining applications to midwifery programmes, high vacancy rates in some geographies, workforce attrition and growing concerns regarding staff well-being have raised questions regarding the sustainability of maternity services.^11–15^

In the UK, these workforce pressures are occurring within a broader context of strain within maternity services and care. Successive national reports and inquiries have highlighted concerns regarding staffing, safety, workplace culture and service sustainability.^16–19^ Policy responses have sought to address these challenges through workforce expansion and service reform initiatives.^20–22^ The National Maternity Review in England ‘Better Births’,^18 19^ similar reports in the devolved nations^23–25^ and subsequent workforce strategies^26 27^ have emphasised the importance of developing a sustainable and supported workforce capable of delivering high quality, personalised maternity care. However, despite persistent policy attention, workforce challenges remain.

Recruitment and retention are often discussed as separate issues,^28^ yet workforce sustainability can be better understood across the professional lifespan, from entry into training and transition into practice to continued participation, movement between roles or settings, career breaks, return to practice and eventual retirement. Recent work has argued that workforce sustainability involves more than maintaining workforce numbers and should instead be understood as sustaining the conditions that enable people to contribute meaningfully and remain within systems over time.^29^ Through this conceptualisation of sustainability, workforce decisions are shaped through interactions across multiple levels, rather than arising from isolated factors.

Individual influences on workforce sustainability may include personal motivations, values, well-being, identity formation and experiences across career transitions. Organisational influences may include workplace cultures, staffing conditions, professional support structures, leadership and opportunities for development. Finally, sociocultural influences encompass wider social expectations, norms (like gender norms), policy environments and cultural understandings of professional work. These dimensions are interrelated: organisational cultures shape experiences of belonging and well-being, while broader societal and institutional structures influence how professional roles are valued and experienced. Understanding recruitment and retention therefore requires attention not only to workforce numbers and service needs but also to the values, relationships and system conditions that shape professional experiences. These interdependencies create complexity, and therefore challenge, for policymakers and service leaders searching for transferable learning that can inform workforce strategies. Existing evidence has therefore proved difficult to translate into actionable strategy for the midwifery workforce.

To explore what was known on the issue of workforce sustainability within midwifery, we conducted an international scoping review in 2026^30^ examining barriers, enablers and interventions associated with midwifery workforce recruitment and retention. This review identified evidence spanning multiple settings and career stages and highlighted recurring themes, including workplace culture, professional identity, well-being, transition experiences, staffing pressures and societal perceptions of midwifery. It also identified a range of proposed strategies and suggested that the same intervention or workforce condition may operate differently across contexts, career stages and workforce groups.

These findings reflect a wider challenge within healthcare workforce research, in that studies frequently identify barriers and enablers associated with recruitment and retention but provide limited explanatory understanding of why or how they shape workforce decisions.^28 31 32^ Existing research often catalogues factors linked to joining or leaving the profession, yet provides less insight into how these influences interact across contexts and career stages.^29^ Similar workforce conditions may therefore be experienced differently depending on organisational culture, professional identity, local workforce structures or wider sociocultural environments.^33 34^

Understanding this complexity, rather than considering determinants in isolation, brings a more nuanced approach to challenges to workforce sustainability. Decisions to enter and remain within the profession emerge through relationships between people and the systems within which they work, and understanding these mechanisms is important if workforce policies are to move beyond short-term responses and support the conditions that enable midwives to join, remain within and contribute meaningfully to the profession over time. It is therefore important to understand why some conditions support recruitment and retention in particular settings, while producing limited impact elsewhere.

### Realist approach

Realist review is a theory-driven approach to evidence synthesis. It aims to explain how and why outcomes occur by examining the interaction between contexts, mechanisms and outcomes.^35 36^ Rather than asking simply whether interventions work, realist approaches seek to understand what works, for whom, in what circumstances and why.

This approach is particularly suited to complex social phenomena in which outcomes emerge through interactions between individuals and their wider environments. Decisions to join and remain within the midwifery profession arise through interactions across multiple levels, including workplace cultures, professional identities, transition experiences, staffing conditions and wider social influences. These factors operate within healthcare systems characterised by substantial contextual variation. Experiences that support retention in one setting may therefore operate differently elsewhere.

Our preceding scoping review^30^ identified substantial heterogeneity within the literature and highlighted context-sensitive relationships, such as the importance of culturally concordant role models for recruitment, the influence of workplace culture and professional autonomy on retention and the vulnerability of the transition-to-practice period. The conventional synthesis approach, employed during the scoping review, was unable to fully explain these relationships but a realist approach will offer an opportunity to move beyond description and develop explanatory programme theory regarding how recruitment and retention strategies operate across contexts. This realist review will seek to develop a programme theory explaining how contextual and causal factors shape workforce sustainability, including whether midwives enter and remain within the profession.

### Study aims and objectives

The overall aim of this realist review is to identify context-mechanism-outcome configurations (CMOCs) capable of explaining how factors influencing workforce sustainability shape whether midwives enter and remain within the profession and under what circumstances these mechanisms become activated.

The objectives are:

1. To identify and develop candidate theories explaining how individual, organisational and sociocultural influences shape recruitment and retention.
2. To examine how contextual factors influence the mechanisms through which workforce decisions around recruitment and retention are shaped.
3. To refine CMOCs that explain how workforce outcomes are generated within midwifery in different settings and across different career stages.
4. To develop a programme theory that will inform UK workforce policy and practice in relation to midwifery.

The review will be guided by the following realist research question:

How, why, for whom and under what circumstances do individual, organisational, and sociocultural factors shape recruitment and retention within the midwifery workforce?

### Workforce sustainability

This review is being conducted within Workforce Voices, a National Institute for Health and Care Research (NIHR)-funded Workforce Research Partnership (NIHR160772). Workforce Voices brings together researchers from five universities across England, with organisational leaders, staff, patients and the public. They have expertise and lived experience spanning healthcare workforce research, public health, maternity care, primary care, clinical education, social and behavioural sciences, organisational psychology, health economics, and human resource management.

Within Workforce Voices, workforce sustainability is understood as the ongoing capacity to sustain conditions that enable people to contribute meaningfully, maintain well-being and continue their work across time and changing contexts. It therefore encompasses workforce capacity, well-being, inclusion, learning and longer-term system resilience.^29^

Within the context of this review, workforce sustainability provides the overarching conceptual lens through which recruitment and retention are understood. Recruitment and retention are seen as interconnected processes that contribute to the long-term capacity of the profession to sustain itself. Decisions to enter, remain within, leave or return to midwifery are shaped by interactions between individuals and the organisational, professional and societal contexts in which they work. Understanding these processes is therefore central to understanding workforce sustainability. A realist approach is particularly valuable, then, because it enables examination of how these influences operate across different contexts and career stages, generating explanatory insights into the conditions that support a sustainable midwifery workforce. By focusing on how and why workforce experiences shape participation in the profession over time, this review seeks to contribute to a more nuanced understanding of workforce sustainability and to inform policies and strategies that support the future of the midwifery workforce.

## Methods and analysis

### Patient and public involvement

Consistent with realist methodology, stakeholder engagement will be embedded throughout the review process to support theory development and refinement.^37^ Realist reviews seek to understand how and why outcomes occur within particular contexts. Stakeholder perspectives are therefore valuable in strengthening explanatory depth and ensuring developing programme theories remain grounded in practice realities and lived experience.

Two complementary Expert Advisory Panels (EAPs) have been established to provide iterative input across the review process. Together, these panels form a central component of the stakeholder infrastructure underpinning the review as illustrated in figure 1. Establishing two panels enables engagement with different forms of expertise and supports examination of workforce sustainability from multiple perspectives.

**Figure 1:**
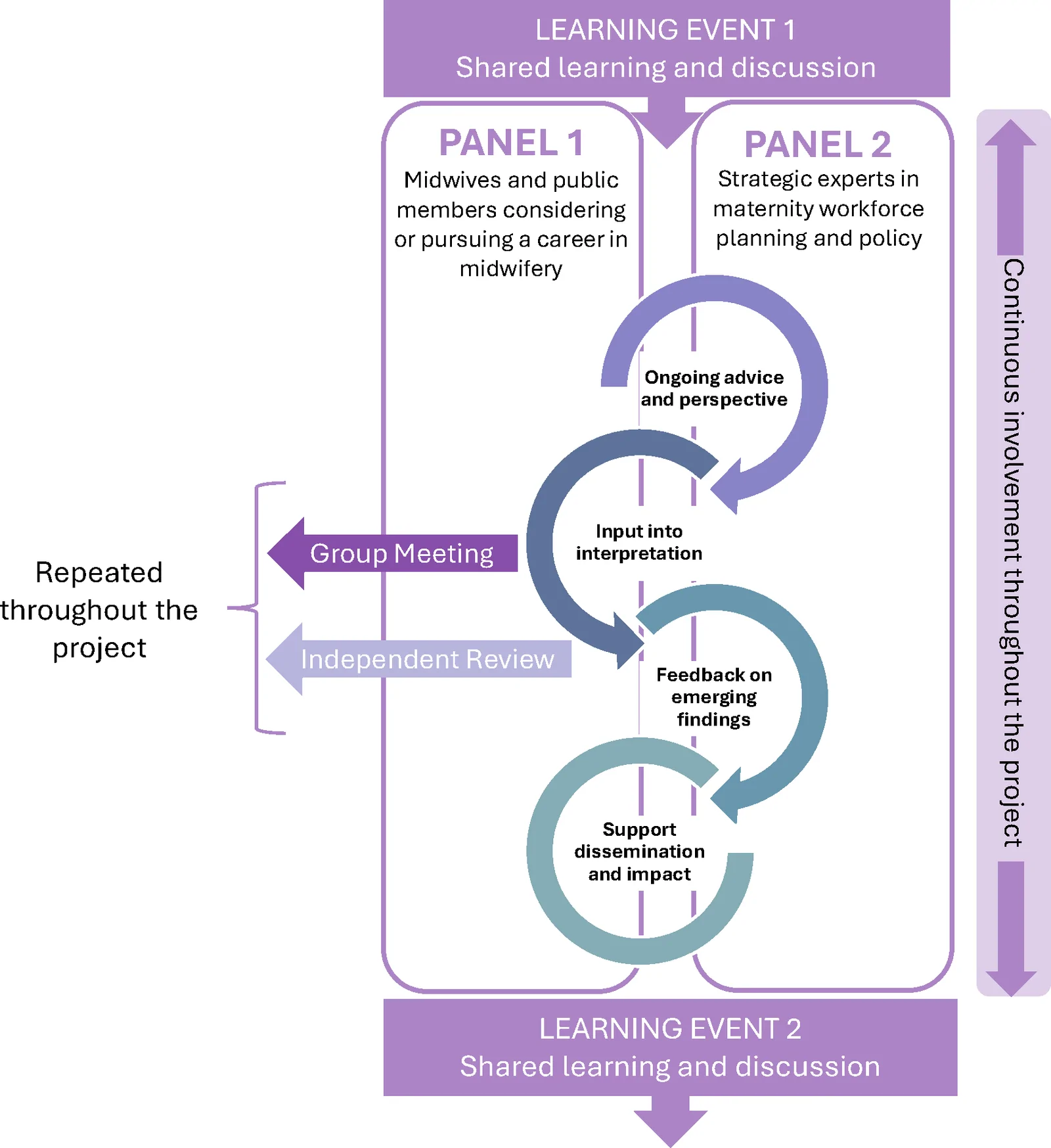
Overview of expert advisory group input across panels 1 and 2.

The first panel comprises practising midwives from different career stages and from different service settings: a student midwife, a former student midwife who left the profession during training, a former practising midwife who subsequently left the profession, and a member of the public considering midwifery as a career. This panel contributes perspectives grounded in day-to-day experiences of entering, training for, leaving and working within the profession. Membership has been purposively developed to capture variation in career stage, professional role and service context, including perspectives from those considering a career in midwifery and those who have left at different stages of the pathway, ensuring representation across different experiences of workforce recruitment and retention. Input will occur through structured meetings (larger arrow figure 1) and independent review activities (smaller arrow figure 1). Participants will be invited to attend up to three meetings, each approximately 1 hour in duration, alongside a small amount of independent review work across the project period.

The second panel comprises senior stakeholders with strategic expertise relevant to maternity workforce planning and policy. Membership includes individuals working in leadership, workforce, educational and organisational roles with knowledge of wider system influences on recruitment and retention alongside patient and public members with experience of maternity services. This panel will contribute perspectives regarding implementation, workforce strategy, service structures and policy contexts. Their input will be more continuous (figure 1) through established Workforce Voices Infrastructure. Both panels will come together at the start and end of the review at a learning event (figure 1) which will also include patients and the public, alongside broader clinical staff groups (eg, obstetric consultant, resident doctors) from across maternity services. Stakeholder involvement has been designed to support specific elements of realist inquiry rather than functioning solely as general consultation. EAPs will contribute focused input at key points throughout the review process to aid interpretation and refinement of programme theory. Contributors will review summaries of emerging findings and provide feedback regarding interpretation and relevance to practice contexts. This iterative process supports theory refinement by bringing explanatory depth and multiple perspectives to emerging analyses.

### Methods

The review will commence in mid-2026 and be completed by early 2027. It will follow a realist approach informed by Pawson’s six iterative stages of realist synthesis (identified in figure 2 as [1]-[6]). The six stages are (1) identifying the review question and initial programme theory (IPT), (2) searching for evidence, (3) appraising evidence, (4) extracting data, (5) synthesising evidence and (6) testing and disseminating the review conclusions. Rather than functioning as a linear sequence of procedural steps, realist inquiry involves ongoing movement between searching, theory development, evidence identification and synthesis as programme theories evolve.^38^

**Figure 2:**
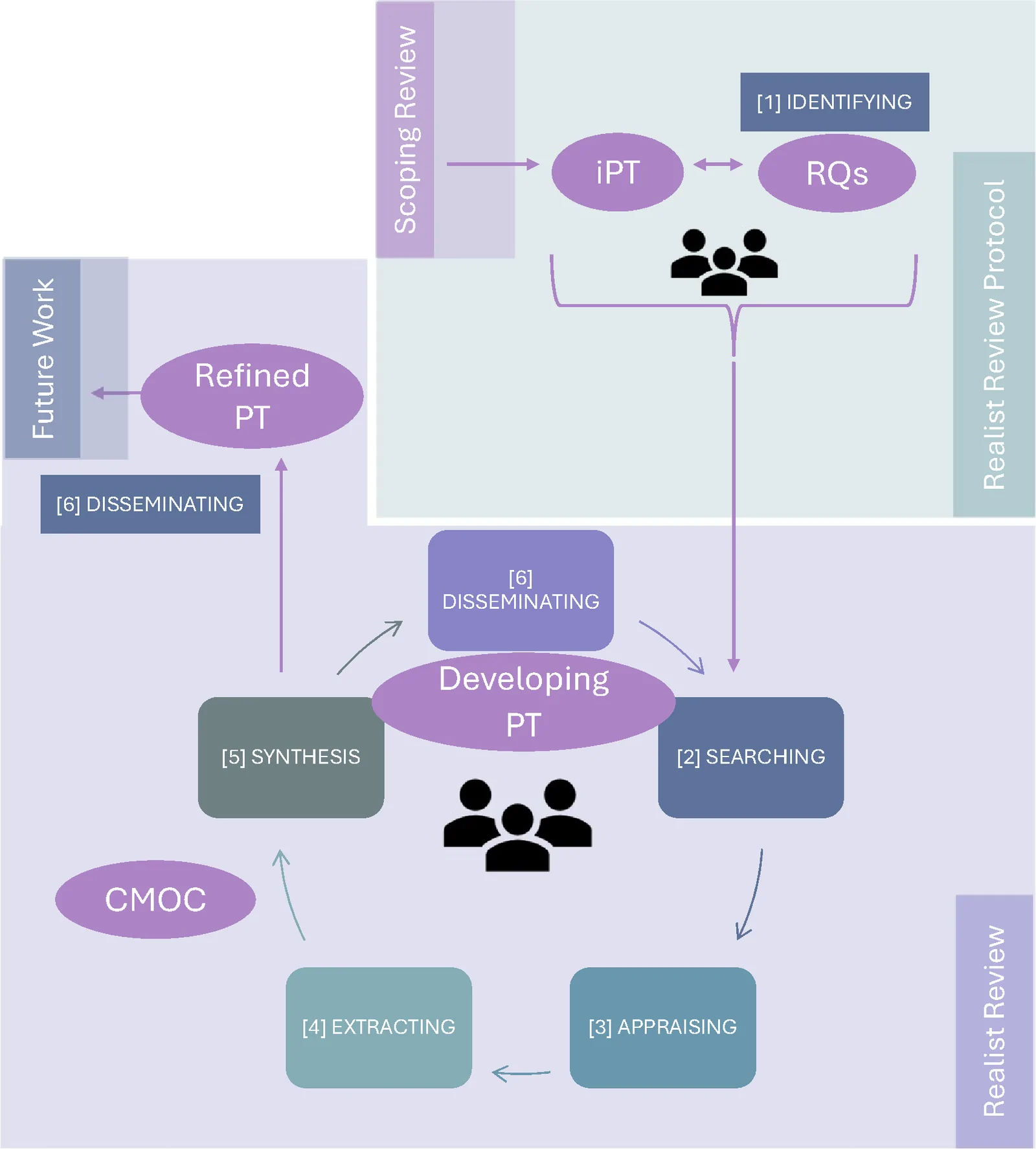
Overview of methods and output alongside the relationship between past, present and future review stages. Numbered boxes related to Pawson’s 6-iterative stages. Oval output boxes: CMOC, context-mechanism-outcome configuration; iPT, initial programme theory; PT, programme theory; RQs, research questions. People icon: stakeholder input.

In this review, a candidate theory is a provisional explanatory proposition about how and why a factor may influence recruitment or retention. Candidate theories were organised within five domains of an IPT, which provides the provisional overarching explanation that will guide searching and synthesis. During synthesis, these propositions will be tested and refined as CMOCs, which describe how particular contexts influence the resources, reasoning or responses through which outcomes are generated.

Figure 2 provides an overview of the methodological process underpinning the review, illustrating the relationship between our earlier scoping review, this protocol, and subsequent realist review phases.

#### Step 1: Identifying programme theories

During protocol development, the realist research question and IPT were developed from the preceding scoping review, stakeholder discussions and relevant conceptual literature. These sources have been used to identify recurring explanatory ideas relating to workforce sustainability, including workplace culture, professional identity, well-being, transition experiences and organisational support. Recurring determinants, intervention strategies and explanatory ideas were expressed as candidate theories and organised within five domains of the IPT. These provisional theories will guide searching and will be tested, challenged and refined throughout the review.

The resulting IPT is presented in the Initial programme theory section.

#### Step 2: Searching

Searching within a realist review differs from conventional systematic review approaches because its purpose is explanatory rather than exhaustive.^36^ Searches are designed to identify data capable of informing, testing and refining programme theory. Figure 3 presents the search strategy across five stages. Although shown as a linear process, searching will proceed iteratively throughout the review in response to programme theory development. Further searches may be undertaken to explore emerging concepts, address gaps in understanding, and refine theoretical explanations identified through stakeholder discussions and synthesis.

**Figure 3:**
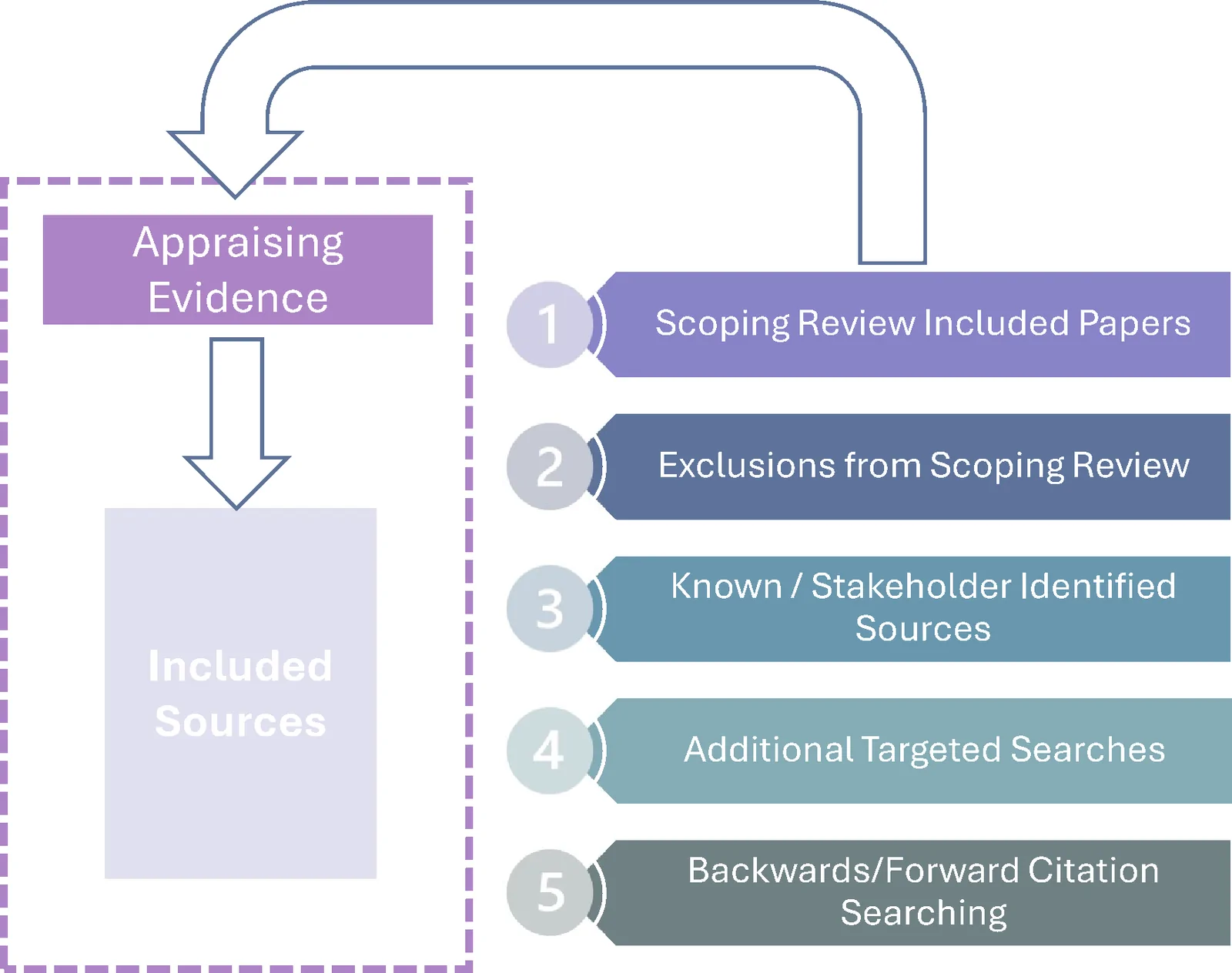
Overview of search strategy.

The preceding scoping review provides the first set of sources for the realist search. Full details of its search are provided in online supplemental file 1. Sources included in that review will be reappraised for relevance to (stage 1), while records excluded because of scoping review-specific publication restrictions will be reconsidered where they may contribute explanatory data (stage 2). Sources identified through the research team and stakeholders (stage 3) will be combined with targeted academic and grey-literature searches driven by emerging theory (stage 4); specific strategies outlined in figure 4 Backward and forward citation searching will be undertaken from relevant sources identified in the preceding stages (stage 5).

**Figure 4:**
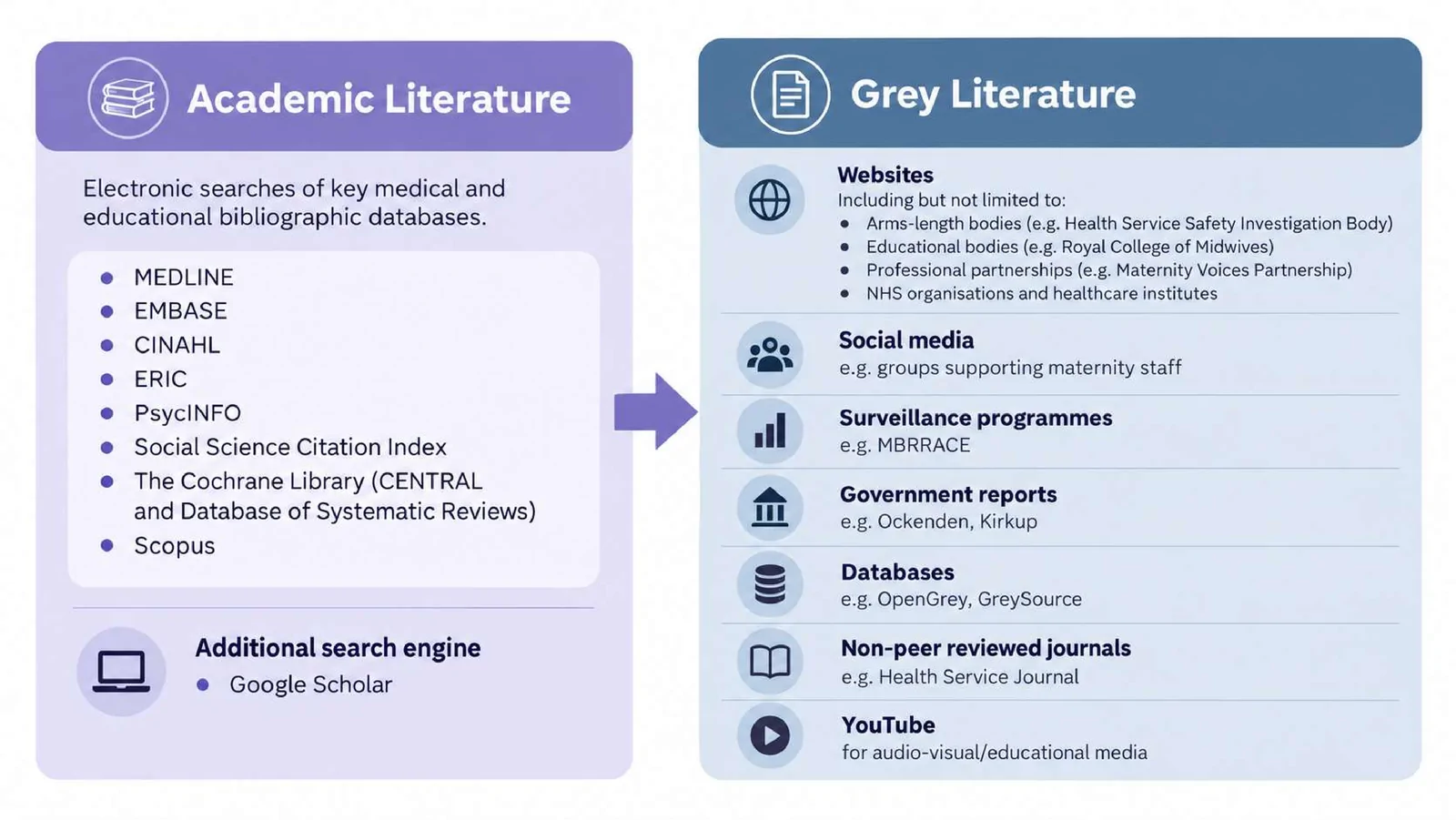
Additional targeted searches.

In keeping with realist methodology no restrictions will be placed on study design, publication format or date of publication. Inclusion will instead be determined by the relevance and contribution of a source to programme theory development, refinement or testing. Older sources may contain important conceptual, theoretical or contextual insights that remain relevant and source diversity including qualitative, quantitative, mixed-methods and grey literature may all contribute explanatory insight. Pragmatically, only English-language material will be included.

#### Step 3: Appraising evidence

In realist synthesis, appraisal focuses on the contribution evidence can make to theory development rather than relying solely on methodological hierarchies.^35 38^ Once the IPT has been established, evidence sources are assessed according to their potential explanatory contribution.^38 39^ Evidence will therefore be appraised according to relevance, richness and rigour using a realist-informed framework (table 1).^35 40^ Relevance refers to the extent to which a source can contribute to answering the realist research questions or refining programme theory.^35 38^ Richness concerns explanatory depth and the degree to which findings illuminate relationships between contexts, mechanisms and outcomes.^35 39^ Rigour will be assessed in relation to the method by which the relevant data were generated and the extent to which claims are supported.^35 38^ For empirical research, assessment will consider the appropriateness and transparency of the methods and the relationship between the data and the claims made. For non-academic and grey literature, it will consider authorship and organisational provenance, transparency of purpose and evidence sources, internal consistency and corroboration with other evidence.

**Table 1:** Realist-informed framework for appraisal of evidence sources according to relevance, richness and rigour. Adapted from Dada *et al*^41 42^

Criterion	Definition	High	Medium	Low
Relevance	Extent to which a source contributes to answering the realist research questions or informs development, testing or refinement of programme theory.	Provides substantial data directly informing candidate theories, CMOCs or programme theory refinement.	Provides some relevant data or contributes indirectly to theoretical development.	Minimal contribution to theory development or realist research questions.
Richness	Degree of explanatory depth and detail provided regarding relationships between contexts, mechanisms and outcomes.	Provides detailed explanatory insight with sufficient contextual detail to support CMOC development or refinement.	Provides partial explanatory insight with some relevant contextual or mechanistic information.	Provides descriptive findings with limited explanatory detail.
Rigour	Credibility of the method by which the relevant data or claim was generated and the extent to which it is adequately supported, judged in relation to the source type.	Provenance and methods or evidence sources are transparent, and the relevant claims are well supported or corroborated.	Some information about provenance or methods is available but reporting or support for the relevant claim is limited.	Provenance or methods are unclear, or the relevant claim is unsupported, internally inconsistent or contradicted by stronger evidence.

CMOC, context-mechanism-outcome configuration.

For this review, evidence will be considered relevant where it contributes to understanding how individual, organisational or sociocultural factors influence the recruitment or retention of the midwifery workforce, or where it provides explanatory insight into the contexts or mechanisms through which workforce sustainability is shaped. Sources need not focus exclusively on recruitment or retention outcomes if they offer theoretically informative evidence capable of developing, testing or refining programme theory. Studies carried forward from the preceding scoping review had previously been selected for relevance to midwifery workforce recruitment and retention, including barriers, enablers and workforce strategies across career stages and healthcare settings. Within the realist review, these sources will be re-appraised according to their explanatory contribution to programme theory development using the above principles of relevance, richness and rigour.

Appraisal is understood as an ongoing analytic activity rather than a fixed procedural checkpoint. Sources may therefore be revisited throughout the review where evolving theories reveal additional explanatory value. Initial appraisal will support decisions regarding inclusion but will continue throughout the review as programme theory develops.

#### Step 4: Extracting data

Data extraction during the protocol phase involved annotation, memo writing and exploratory notetaking to support candidate theory development. This approach aligns with Pawson’s original conceptualisation of realist review, where early extraction is oriented towards identifying explanatory fragments rather than applying predetermined coding structures.^38^

During the formal review stage, extraction procedures will become more structured. Full texts selected for inclusion will be reviewed in detail and descriptive characteristics including author, publication year, study design and source type recorded in a purpose-designed spreadsheet.

Data relevant to theory development will then be extracted and organised according to their contribution to contexts, mechanisms, outcomes and configurational relationships. Extraction will retain an iterative character throughout the review. As programme theories evolve, previously reviewed papers may be revisited to identify material that acquires new theoretical relevance.

#### Step 5: Synthesising evidence

Synthesis within realist review seeks to explain how outcomes are generated and why these differ across contexts. During protocol development, preliminary synthesis focused on organising findings from the scoping review into candidate theories and five domains within an IPT to guide subsequent realist inquiry.

Formal realist synthesis will occur across two interconnected levels. The first level involves engagement with empirical material and development of explanatory configurations. This process may include juxtaposing findings from different studies, reconciling apparently contradictory findings, adjudicating between competing explanations and identifying patterns across contexts.^38^ Interpretive cross-case comparison will support analysis of how particular contexts shape responses to interventions and produce differing outcomes. Explanatory insights may therefore be assembled across multiple documents. Mechanisms inferred from one source may assist interpretation of outcomes observed elsewhere. Extracted material will contribute to testing, refining or rejecting emerging CMOCs. Ongoing team discussions and stakeholder engagement will support reflexive interpretation and programme theory development.

The second level of synthesis involves theoretical integration. As individual configurations are refined and explanatory patterns become clearer, CMOCs will be brought together into a revised programme theory capable of explaining workforce sustainability across contexts. Programme theory development will continue iteratively until sufficient explanatory coherence is achieved. Consistent with realist principles, explanatory adequacy will be judged according to criteria including explanatory breadth, coherence and alignment with existing knowledge.^35 38^

#### Step 6: Dissemination

Step 6 will involve drafting and testing the review’s emerging explanations and conclusions with stakeholders before wider dissemination. The research team and Expert Advisory Panels will review the developing programme theory and proposed conclusions for explanatory plausibility and relevance across practice contexts. Feedback may prompt further searching or refinement before the final programme theory is agreed.

The final programme theory and review conclusions will then be disseminated through peer-reviewed publication, conference presentation and outputs developed for professional, policy and stakeholder audiences. The detailed dissemination plan is provided in the Dissemination section.

### Initial programme theory

The IPT presented here should be understood as a provisional explanatory framework developed from patterns identified within the preceding scoping review and stakeholder discussion. It represents a set of hypotheses to guide realist inquiry rather than an explanation generated through realist synthesis and will be iteratively tested and refined during the review.

The IPT comprises five overlapping domains: career pathways, societal and professional influences, workplace environment, access and resources and well-being. It proposes that, across the professional trajectory, structural, organisational and professional conditions shape the resources available to people and their perceptions of feasibility, safety, belonging, professional value and well-being. Supportive conditions may strengthen confidence, professional identity and capacity to participate, supporting entry, continued participation or return to midwifery. Adverse conditions may constrain entry or contribute to movement out of roles or the profession. These relationships are expected to vary according to career stage and setting and will be specified and tested as CMOCs during the review.

The IPT (figure 5) situates recruitment and retention within the broader concept of workforce sustainability. It represents workforce participation as a dynamic and non-linear process extending across the professional lifespan, from early career contemplation to retirement. Central to figure 5 is the retention spectrum, which illustrates how individuals may move into, within, out of, and back into the workforce at multiple stages of their careers. Recruitment is therefore understood not as a single event, but as occurring at several points across the trajectory, including application to midwifery degree programmes, transition into employment, movement between organisations, return to practice and progression into specialist or leadership roles. The dashed elements within the figures represent this permeability of workforce participation and the potential for midwives to re-enter or alter their engagement with the profession over time.

**Figure 5:**
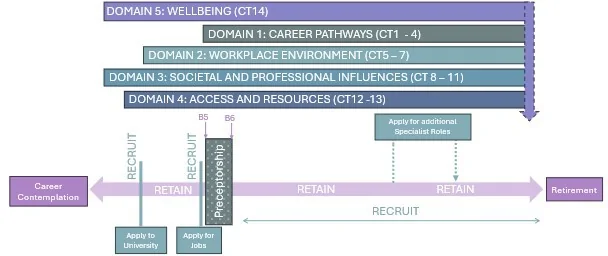
Domains of the initial programme theory across the midwifery workforce trajectory. B5, Band 5 midwife (newly qualified); B6, Band 6 midwife (post-preceptorship/independent practitioner); preceptorship, structured period of supported practice for newly qualified midwives.

Key transition points are highlighted within the spectrum, including entry into training and into work via the preceptorship programme—ensuring newly qualified Band 5 midwives have the Band 6 competencies they need to practise independently—and onto senior roles. These transition periods are theorised as particularly important in shaping workforce participation, as they may either strengthen commitment and professional identity or contribute to disengagement and attrition. Throughout this review, Band 5 and Band 6 are used as shorthand for stages of professional development and expected levels of practice; while these bands are formally linked to NHS pay scales, they are also commonly used within maternity services to denote differences in experience, responsibility and capability.

Figure 5 demonstrates the interrelationship within domains of the IPT and across the retention spectrum. While each domain of the IPT represents a distinct area, they overlap considerably in practice. well-being is shown as intersecting all domains of the IPT, in addition to standing alone, reflecting its role as both an independent influence on retention and a mechanism through which workplace, professional, societal and structural factors are experienced. Each domain houses a number of candidate theories (table 2) developed from the preceding scoping review. The density and overlap of the candidate theories reflect the complexity of recruitment and retention within midwifery, where multiple contextual influences and mechanisms operate concurrently and interact dynamically across individual, organisational and societal levels. In keeping with realist methodology, they are intended as initial explanatory propositions that will be iteratively refined throughout the review process.

**Table 2:** Domains and candidate theories within the initial programme theory

Domain no.	Domain label	CT no.	CT label	Candidate theory summary
**1**	Career pathways	1	Transition into workforce	Recruitment and retention may improve when transition into practice is supported, expectations are realistic and new midwives feel prepared and welcomed into the workforce.
		2	Preceptorship and early career	Early-career support and protected preceptorship may help midwives develop confidence, professional identity and commitment to remaining in practice.
		3	Workforce structure and career development	Opportunities for career progression and meaningful professional development may encourage midwives to remain within the profession.
		4	Seniority	Retaining experienced midwives and supporting progression into senior roles may stabilise the workforce and reduce pressures on early-career staff.
**2**	Societal and professional influences	5	Gendered and societal norms	Gender norms, cultural expectations and structural inequalities may influence who is able to enter, remain and progress within midwifery.
		6	Perceptions of the profession	Recruitment and retention may be influenced by whether midwifery is perceived as valued, meaningful and sustainable or as stressful and undervalued.
		7	Professional identity	Midwives may be more likely to remain in practice when workplace environments support autonomy, woman-centred care and alignment with professional values.
**3**	Workplace environment	8	Perceptions of training	Experiences of education and training may shape confidence, preparedness, belonging and long-term engagement with the profession.
		9	Staffing, resources and workload	Staffing levels, workload and resource availability may influence well-being, perceptions of safety and decisions to remain in practice.
		10	Workplace culture and treatment	Supportive workplace cultures characterised by respect, inclusion and positive leadership may improve retention, while bullying and discrimination may contribute to attrition.
		11	Rural and conflict settings	Recruitment and retention in rural or high-risk settings may depend on the balance between environmental challenges, organisational support and community connection.
**4**	Access and resources	12	Financial and structural access	Financial pressures and structural barriers may affect access to midwifery education and influence workforce diversity and recruitment.
		13	Salary and financial	Pay, financial security and employment conditions may shape perceptions of professional value and influence retention.
**5**	Well-being	14	Well-being	Midwives may be more likely to remain in practice when work is compatible with personal well-being, work–life balance and the ability to provide meaningful care.

CT, candidate theory.

## Ethics and dissemination

### Ethics

The review involves analysis and synthesis of existing published and publicly available material and does not involve the collection of primary research data. Formal ethical approval was therefore not required.

### Dissemination

Findings from this review will be disseminated through peer-reviewed publication, conference presentations and outputs tailored for stakeholder audiences. Dissemination activities will include academic and professional audiences involved in maternity care, workforce planning and policy development. Accessible summaries will also be developed with stakeholder input to support wider dissemination and enhance relevance for maternity workforce communities. Findings will contribute to the development of evidence-informed recommendations relating to midwifery workforce recruitment and retention and may inform future workforce policy and service initiatives.

## Supplementary material

10.1136/bmjopen-2026-124745 online supplemental file 1

## Data Availability

Data are available upon reasonable request.
