# Supplementary material for: Understanding midwifery workforce recruitment and retention: a realist review protocol for synthesising international evidence to inform UK workforce strategy
Source: BMJ Open. 2026 Oct 20;16(10):e124745. doi: 10.1136/bmjopen-2026-124745 (PMC13684146; doi:10.1136/bmjopen-2026-124745)
Supplement: Supplementary file 1 [file bmjopen-2026-124745.reviewer_comments.pdf]

PEER REVIEW HISTORY

BMJ Open publishes all reviews undertaken for accepted manuscripts. Reviewers are asked to complete a checklist review form and are provided with free text boxes to elaborate on their assessment. These free text comments are reproduced below.

ARTICLE DETAILS

Title (Provisional)

Understanding Midwifery Workforce Recruitment and Retention: A Realist Review Protocol for Synthesising International Evidence to Inform UK Workforce Strategy

Authors

Stelling, Heidi; Brown, Megan E L; Boyle, Alex; Higgins, Christina; Dodd, Chris; Todd, Erin; Hoverd, Eleanor; Park , Sophie; Burford, Bryan; Vance, Gillian

---

VERSION 1 - REVIEW

**Reviewer**	**1**
**Name**	**Long, Jaqui**
**Affiliation**	**The University of Sheffield, SCHARR**
**Date**	**03-Aug-2026**
**COI**	**None**

Overall, I think your review has clear aims and is largely well described. I have put a lot of detailed comments in the attached document. The diagrams need improving for clarity and I think the protocol could be a bit shorter as there is some repetition. Realist methodology and process is difficult to describe and I think some definition of terms would be helpful. Good luck with the review!

Thank you for the opportunity to review this manuscript. Overall the document is well written, although it does seem to be rather repetitive in places and I think could be shorter without losing meaning and clarity. Also the use of commas is at times confusing to me and seems unnecessary e.g. ‘should, rather, be understood’ – this would read more clearly without the commas. Below are my specific comments:

- There are no dates of the study, which the review requirements say should be present. Please add these.
- Keywords – I suggest adding ‘Review’ to these
- Abstract, Methods, second line – suggest this should read ‘are informed’ rather than ‘were informed’
- Strengths and limitations, second point – suggest ‘creating’ rather than ‘providing’

- Introduction, p3, para 1, line 4 – give some examples of these wider health system benefits.
- Intro, p3, para 1, line 5 – I think this should read 'including increasingly', to make it clear the UK is part of the world!
- Intro, p3 second paragraph – make it clear that this relates to the UK rather than more widely.
- P4, para 2, first sentence – not everyone will progress during their career but they may still stay in the profession – this seems to be missing from this definition.
- P4, para 3, first line – can you add a date to 'recently' for clarity
- P5, para 1, 5th line – think this should read 'responses and' rather than 'to'
- P5, para 4, line 5 – there is a capital A
- P5, para 4, line 8, think this should read 'influence workforce sustainability and'
- P6, first line – professional hasn't been previously included as a specific influence separate from the others – maybe best to keep consistency with the 3 factors?
- P6, 1.3, para 1, line 3 – I think the 'from 5 universities' needs to come after the 'researchers' as the other participants are not part of the universities. Also should be plural patients.
- P6, 1.3 – suggest combining paras 2 and 3
- P6, 1.3, para 3, line 2 – suggest 'and that support'
- P7, para 4 – was any consideration given to including midwives who have left the profession? This would seem to be a valuable additional perspective.
- P7, para 8 – can you clarify who the patient and public members are a bit more? The combination of senior roles and patients at the moments seems a bit strange.
- P8, para 1 – suggest putting the service users before the 'broader clinical staff group'. Also you have referred to them as patients up to now – suggest consistent terminology.
- P8, figure 1 – the columns need to be wider to avoid losing the numbers next to word panel, or put the number on the next line.
- P8, Methods – suggest a brief description of the six stages, could be in the form of a Figure?
- P8, Methods – I think it would be useful to have some definitions of terminology used within this including Candidate Theory, IPT, CMOCs. Also, having introduced the abbreviations, these are often then not used later on, which is confusing.
- P8, Methods, last line and p9 first para – this would go better if it was integrated into the Step 1 section on page 9
- P9 second para – terminology moves between multiple IPTs and a single IPT – this is confusing, suggest consistently talking about multiple IPTs at this stage.

- P9 Figure 2 – I find this diagram very confusing and unhelpful. The colours also do not translate well into black and white. I think this needs reworking. It's not clear who the people in the middle are, and there are lot of abbreviations.
- P10, second para – suggest removing the references to the other sections, as these are confusing.
- P10, Step 2, second para – the first sentence belongs in Step 1.
- P10, Step 2, second para, last sentence. This links to Figure 3, where I think it would be better for the backward/forward searching to be put at the end of the sequence rather than in the middle.
- P11, fig 3 – colours in the diagram don't show up well in black and white. See previous comment about sequence. The sentence in italics below the figure doesn't provide clarity – suggest removing it.
- P11 – Box 1 needs to be referenced in the text rather than in the sentence with Fig 3. Could it be called a figure rather than a box so there aren't three types of diagram?
- P12, Step 3 para 1, last sentence – how will rigour be judged with the non-academic literature?
- P13, Step 5, para 1 – again, suggest keeping with multiple IPTs rather than single.
- P14, Step 6, most of first two paras is not dissemination and needs to be put elsewhere or cut if it duplicates other sections.
- P14, section 2.3 - I wonder if this whole section would be better integrated into step 1 rather than being a separate section?
- P14, section 2.3, para 2 – this should be Figure 4. Also, the language is now about recruitment and retention but you have been talking about sustainability up to now – suggest keeping this consistent.
- P15, figure 4 – I don't find the dashed lines communicate what you are trying to indicate – maybe needs another approach? I'm also not sure about having Wellbeing as a cross cutting theme in this way, as this seems to place it as a particular place on the timeline. Is there a way of perhaps making it run along the whole length of the diagram, or be a box encompassing the other 4? Also, the words in brackets on each IPT in the diagram are not readable, as the text seems to have been compressed in some way.
- P16, and more broadly. I am not clear what your initial IPTs are – the labels don't really clarify this as they are very broad. Do you already have provisional CMOCs for each of these? If so, it would be good to include these.
- P16, Ethics – I think this needs to be earlier in the document, unless it has been specified it should be at the end.
- P17, Dissemination – you already have this as step 6 in the process so this is duplication, maybe remove this section?

VERSION 1 - AUTHOR RESPONSE

Reviewer 1

No.	Comment	Action	Pg No.
1	From summary: The diagrams need improving for clarity and I think the protocol could be a bit shorter as there is some repetition. Realist methodology and process is difficult to describe and I think some definition of terms would be helpful.	Thank you for these helpful comments. We have addressed each of the points raised. The diagrams have been revised to improve clarity, repetition throughout the protocol has been reduced, and additional definitions and clarification of key realist methodology terms have been incorporated to support readers who may be less familiar with the approach.	Throughout
2	Thank you for the opportunity to review this manuscript. Overall the document is well written, although it does seem to be rather repetitive in places and I think could be shorter without losing meaning and clarity. Also the use of commas is at times confusing to me and seems unnecessary e.g. 'should, rather, be understood' – this would read more clearly without the commas.	Thank you. We have revised the manuscript throughout to reduce repetition and simplify sentence structure. In particular, we have condensed the discussion of workforce sustainability in section 1.3, consolidated repeated descriptions of initial theory development within Step 1, and revised Step 6 to distinguish the realist process of testing conclusions from the dissemination plan in section 3.2. We have also reviewed comma use throughout and removed unnecessary interrupting commas.	Throughout
3	Below are my specific comments:<br>- There are no dates of the study, which the review requirements say should be present. Please add these.	Thank you for highlighting this. We have added approximate dates for the review, with the review anticipated to commence in mid-2026 and be completed by early 2027. As the date of protocol publication is not yet known, this has not been specified. The anticipated completion date is also necessarily approximate, as the duration of the review will depend on the volume and complexity of the available	2<br>9

		evidence and the iterative nature of the realist synthesis.<br><br>The dates have been added to the Abstract (Methods) and the main Methods section.	
4	- Keywords – I suggest adding 'Review' to these	Added.	2
5	- Abstract, Methods, second line – suggest this should read 'are informed' rather than 'were informed'	Revised.	2
6	- Strengths and limitations, second point – suggest 'creating' rather than 'providing'	Thank you for this suggestion. In line with the Editor's broader comment, the Strengths and Limitations section has been substantially revised and this wording has therefore been removed.	3
7	- Introduction, p3, para 1, line 4 – give some examples of these wider health system benefits.	Thank you for this helpful suggestion. We have revised the sentence to provide brief examples of the wider health system benefits described in the cited literature.	3
8	- Intro, p3, para 1, line 5 – I think this should read 'including increasingly', to make it clear the UK is part of the world!	Thank you. We have revised the sentence to clarify that the UK forms part of the wider international pattern of midwifery workforce shortages.	3
9	- Intro, p3 second paragraph – make it clear that this relates to the UK rather than more widely.	Amended.	4
10	- P4, para 2, first sentence – not everyone will progress during their career but they may still stay in the profession – this seems to be missing from this definition.	Thank you. We agree that 'career progression' could be interpreted as advancement into more senior roles. We have revised the sentence to describe participation across the professional lifespan, including continued practice without advancement, movement between roles or settings, career breaks and return to practice.	4
11	- P4, para 3, first line – can you add a date to 'recently' for clarity	Added.	5
12	- P5, para 1, 5th line – think this should read 'responses and' rather than 'to'	Amended.	5
13	- P5, para 4, line 5 – there is a capital A	Amended.	5
14	- P5, para 4, line 8, think this should read 'influence workforce sustainability and'	Thank you. We have rephrased the sentence to clarify the	6

		relationship between contextual and causal factors, workforce sustainability, and decisions to enter and remain within midwifery.	
15	- P6, first line – professional hasn't been previously included as a specific influence separate from the others – maybe best to keep consistency with the 3 factors?	Thank you. We have removed 'professional' as a separate level of influence from Objective 1 to maintain consistency with the individual, organisational and sociocultural levels used elsewhere in the manuscript and in the realist research question. Professional identity, values and professional influences remain included within the substantive candidate theories.	6
16	- P6, 1.3, para 1, line 3 – I think the 'from 5 universities' needs to come after the 'researchers' as the other participants are not part of the universities. Also should be plural patients.	Amended.	6
17	- P6, 1.3 – suggest combining paras 2 and 3	Amended.	6-7
18	- P6, 1.3, para 3, line 2 – suggest 'and that support'	Thank you. We have revised the sentence to clarify that workforce sustainability concerns the conditions that enable meaningful contribution, wellbeing and continued participation over time.	7
19	- P7, para 4 – was any consideration given to including midwives who have left the profession? This would seem to be a valuable additional perspective.	Thank you for highlighting the value of including perspectives from those who have left the profession. This was considered in constituting the panel, which includes both a former student midwife who left during training and a former practising midwife who subsequently left the profession. We have revised the manuscript to make these perspectives explicit and to clarify that the panel captures experiences of leaving midwifery at different stages of the career pathway.	8
20	- P7, para 8 – can you clarify who the patient and public members are a bit	Thank you for highlighting this. We agree that the description	8

	more? The combination of senior roles and patients at the moments seems a bit strange.	was not sufficiently clear. This section has been rewritten to clarify the roles and contributions of the patient and public members, and to distinguish these from the senior clinical and academic members of the team.	
21	- P8, para 1 – suggest putting the service users before the 'broader clinical staff group'. Also you have referred to them as patients up to now – suggest consistent terminology.	Thank you for this helpful suggestion. We have revised the sentence to place patients and the public before the broader clinical staff group and have used the term 'patients and the public' consistently throughout the manuscript.	8
22	- P8, figure 1 – the columns need to be wider to avoid losing the numbers next to word panel, or put the number on the next line.	Thank you for this comment. We have reviewed Figure 1 and confirmed that the numbers remain visible in the current version. To further reduce the risk of the labels being obscured, we have repositioned and centralised them within the relevant panels.<br><br>We have also clarified in the accompanying text that the overlapping concentric circles are intentional: they illustrate that these individuals engage with the wider workforce voices structure beyond the specific panel shown.	10
23	- P8, Methods – suggest a brief description of the six stages, could be in the form of a Figure?	Thank you. We have added a concise description of all six stages at the beginning of the Methods section. Figure 2 continues to show their relationship within the wider review process, while the subsequent subsections provide the detailed account of each stage. We therefore did not add a further figure or table.	9-10
24	- P8, Methods – I think it would be useful to have some definitions of terminology used within this including Candidate Theory, IPT, CMOCs. Also, having introduced the abbreviations, these are	Thank you. We have added brief definitions of candidate theory, initial programme theory and context-mechanism-outcome configuration at the beginning of the Methods section. We have	10<br>Throughout

	often then not used later on, which is confusing.	also reduced and standardised the abbreviations used throughout: the manuscript now refers consistently to one initial programme theory (IPT), and abbreviations are used only after their first definition.	
25	- P8, Methods, last line and p9 first para – this would go better if it was integrated into the Step 1 section on page 9	Thank you. We have moved the material describing development of the realist research question, candidate theories and initial programme theory from the general Methods introduction into Step 1. We have consolidated it with the existing Step 1 material to reduce repetition. The opening of section 2.2 now provides only a brief overview of the review process and Figure 2.	10-11
26	- P9 second para – terminology moves between multiple IPTs and a single IPT – this is confusing, suggest consistently talking about multiple IPTs at this stage.	Thank you. We agree that the previous alternation between a single and multiple initial programme theories was confusing. We have revised the terminology throughout to describe one initial programme theory comprising five domains, with candidate theories nested within those domains. This terminology is now used consistently in the abstract, Methods, Figure 4 and Table 2.	2<br>9<br>Figure 4<br>Table 2
27	- P9 Figure 2 – I find this diagram very confusing and unhelpful. The colours also do not translate well into black and white. I think this needs reworking.<br>It's not clear who the people in the middle are, and there are lot of abbreviations.	Thank you for these helpful comments. We apologise that the previous version of the figure was not sufficiently clear. We have substantially revised Figure 2, including clarifying the role of the stakeholder group represented in the centre of the figure, revising the text within the diagram, and reducing ambiguity around the abbreviations. We have also added a footnote explaining the figure and defining the abbreviations used.<br><br>As BMJ Open is an online-only journal and publishes figures in	12

		colour, we have retained the use of colour. However, we would of course be happy to make any further changes to improve accessibility if requested during the editorial process.	
28	- P10, second para – suggest removing the references to the other sections, as these are confusing.	Removed.	10
29	- P10, Step 2, second para – the first sentence belongs in Step 1.	Thank you. We have moved the statement concerning the contribution of the preceding scoping review into Step 1, where it forms part of the explanation of initial theory development. We have revised the Step 2 paragraph to focus specifically on how sources from the scoping review provide the starting set of evidence for the realist search and how these are supplemented through citation, stakeholder-informed and targeted searches.	11-12
30	- P10, Step 2, second para, last sentence. This links to Figure 3, where I think it would be better for the backward/forward searching to be put at the end of the sequence rather than in the middle.	Thank you for identifying this. The text has been corrected to reference Figure 3, and the figure has been revised so that the backward and forward searching is positioned at the end of the sequence, as suggested.	13-14
31	- P11, fig 3 – colours in the diagram don't show up well in black and white. See previous comment about sequence. The sentence in italics below the figure doesn't provide clarity – suggest removing it.	Thank you for this helpful comment. The sequence within Figure 3 has been revised as suggested, and the corresponding text in the manuscript has been updated accordingly. We have also removed the italicised sentence beneath the figure, as recommended.<br><br>As noted above, BMJ Open is an online-only journal and publishes figures in colour, so we have retained the colour formatting. We would, however, be happy to make any further changes to improve accessibility	12

		if requested during the editorial process.	
32	- P11 – Box 1 needs to be referenced in the text rather than in the sentence with Fig 3. Could it be called a figure rather than a box so there aren't three types of diagram?	Thank you for this suggestion. Box 1 has now been presented as Figure 4 and is explicitly referenced in the main text. This also provides greater consistency in the presentation and terminology of the manuscript's diagrams.	14
33	- P12, Step 3 para 1, last sentence – how will rigour be judged with the non-academic literature?	Thank you. We have expanded the description of rigour to explain how it will be assessed across different source types. For empirical research, appraisal will consider methodological transparency and the relationship between data and claims. For non-academic and grey literature, it will consider provenance, purpose, transparency of evidence sources, internal consistency and corroboration. We have also revised the rigour row in Table 1 so that the criteria apply to the particular data used in the synthesis rather than only to academic studies.	14-16
34	- P13, Step 5, para 1 – again, suggest keeping with multiple IPTs rather than single.	Thank you. As described in our response to Comment 26, we have revised the manuscript to refer consistently to one initial programme theory comprising five domains. Step 5 has been amended accordingly.	Throughout
35	- P14, Step 6, most of first two paras is not dissemination and needs to be put elsewhere or cut if it duplicates other sections.	Thank you. We agree that the purpose of the first two paragraphs was not sufficiently clear. We have revised Step 6 to explain that emerging explanations and conclusions will be tested with the research team and stakeholders before wider dissemination, with further searching or refinement undertaken where required. We have also reduced repetition with section 3.2, which provides the fuller account of the planned dissemination outputs.	16

36	- P14, section 2.3 - I wonder if this whole section would be better integrated into step 1 rather than being a separate section?	Thank you. We considered integrating section 2.3 into Step 1. We have revised Step 1 to explain more clearly how the IPT was developed and have added a direct signpost to section 2.3. We have retained section 2.3 as a separate section because Step 1 describes the methodological process, whereas section 2.3 presents the substantive provisional theory that will be tested and refined during the review. We believe this separation makes the distinction between process and output clearer.	11<br>16-19
37	- P14, section 2.3, para 2 – this should be Figure 4. Also, the language is now about recruitment and retention but you have been talking about sustainability up to now – suggest keeping this consistent.	Thank you. We agree that recruitment and retention and workforce sustainability were sometimes used too interchangeably. We have revised the manuscript to use workforce sustainability for the overarching conceptual construct and recruitment and retention for the component processes through which people enter, remain within, leave or return to midwifery. The text and Figure 4 title have been amended accordingly.	Throughout
38	- P15, figure 4 – I don't find the dashed lines communicate what you are trying to indicate – maybe needs another approach? I'm also not sure about having Wellbeing as a cross cutting theme in this way, as this seems to place it as a particular place on the timeline. Is there a way of perhaps making it run along the whole length of the diagram, or be a box encompassing the other 4? Also, the words in brackets on each IPT in the diagram are not readable, as the text seems to have been compressed in some way.	Thank you for these helpful suggestions. We have revised the figure substantially in response. The text around the dashed lines have been edited to give a clearer visual approach, and Wellbeing has been repositioned to reflect its standalone role and its influence across all four domains, rather than appearing as a discrete point on the timeline. We have also revised the text in brackets within each IPT to improve readability. We apologise that this text was not sufficiently legible in the previous version and have re-uploaded the revised figure.	20

		Please note that this figure is now numbered Figure 5, following the reorganisation of the figures in the manuscript, with the previous Box 1 now presented as Figure 4.	
39	- P16, and more broadly. I am not clear what your initial IPTs are – the labels don't really clarify this as they are very broad. Do you already have provisional CMOCs for each of these? If so, it would be good to include these.	Thank you. We agree that the previous labels were too broad to function as separate initial programme theories. We have revised the manuscript, Figure 4 and Table 2 to present a single initial programme theory comprising five domains, with candidate theories nested within each domain. We have also added a narrative statement explaining the overarching provisional theory into section 2.3. The candidate-theory summaries are preliminary explanatory propositions rather than completed CMOCs, and we have made this distinction explicit. CMOCs will be developed, tested and refined through the formal synthesis and are therefore not presented prematurely in the protocol.	16-19
40	- P16, Ethics – I think this needs to be earlier in the document, unless it has been specified it should be at the end.	Thank you for this suggestion. We have retained the Ethics and Dissemination sections in their current position in accordance with the journal's protocol article requirements and on the Editors advice.	-
41	- P17, Dissemination – you already have this as step 6 in the process so this is duplication, maybe remove this section?	Thank you for this observation. We agree that dissemination is addressed within Step 6 of the realist review process; however, this describes dissemination specifically from a realist perspective and forms part of the review process. The separate 'Dissemination' section describes the planned dissemination of the study findings in the conventional	-

		sense and therefore serves a different purpose. In addition, the journal requires a subsection titled 'Ethics and dissemination' within protocol articles. We have therefore retained this section, in line with the journal requirements and following confirmation from the Editor.	
