# Supplementary material for: Understanding midwifery workforce recruitment and retention: a realist review protocol for synthesising international evidence to inform UK workforce strategy
Source: BMJ Open. 2026 Oct 20;16(10):e124745. doi: 10.1136/bmjopen-2026-124745 (PMC13684146; doi:10.1136/bmjopen-2026-124745)
Supplement: Supplementary file 2 [file bmjopen-16-10-s001.docx]

Search Strategy

Scoping Review: Recruitment and Retention of Midwifes

# Medline

| **Ovid MEDLINE(R) and In-Process, In-Data-Review & Other Non-Indexed Citations 1946 to June 24, 2025**  **Accessed via ovid.com \| Run Date: 25^th^ June 2025** | | |
| --- | --- | --- |
| **Line#** | **Search String** | **No.** |
| 1  Population: Midwife | midwifery/ or maternal-child nursing/ or obstetric nursing/ or nurse midwives/ or ("birth assistant*" or "birth attendant*" or (midwi?e* adj3 (staff or workforce or personnel or student or undergraduate))).ti,ab,kf. | 34929 |
| 2  Concept: Recruitment and Retention | personnel management/ or job application/ or personnel loyalty/ or personnel selection/ or personnel turnover/ or work engagement/ or employment/ or career mobility/ or return to work/ or unemployment/ or career choice/ or absenteeism/ or presenteeism/ or sick leave/ or exp workforce/ or (hiring or hire or "employment policy" or recruitment or staffing or "staff shortages" or attrition or "employee longevity" or retention or ((staff or workforce) adj3 sustainability) or (workforce adj3 stability)).ti,ab,kf. or ("intention to leave" or "intention to stay").mp. | 640331 |
| 3 | 1 AND 2 | 2534 |
| 4 | Limit to English Language | 2297 |
| 5 | Limit to Year > 2016 | 737 |

# Embase

| **EMBASE 1974 – 2025 June 24**  **Accessed via ovid.com \| Run Date: 25^th^ June 2025** | | |
| --- | --- | --- |
| **Line#** | **Search String** | **No.** |
| 1  Population: Midwife | exp midwife/ or obstetrical nursing/ or (“maternal-child nursing” or birth assistant* or birth attendant* or (midwi*e* adj3 (staff or workforce or personnel or student or undergraduate))).ti,ab,kw. | 44680 |
| 2  Concept: Recruitment and Retention | personnel management/ or job finding/ or staff recruitment/ or work engagement/ or employment/ or career mobility/ or return to work/ or work resumption/ or unemployment/ or absenteeism/ or presenteeism/ or medical leave/ or exp workforce/ or ("job application" or "personnel selection" or "career choice" or "employment policy" or hiring or hire or recruitment or staffing or "staff shortages" or attrition or "employee longevity" or retention or ((staff or workforce) adj3 sustainability) or (workforce adj3 stability)).ti,ab,kw. or ("intention to leave" or "intention to stay").mp. | 875036 |
| 3 | 1 AND 2 | 3507 |
| 4 | Limit to English Language | 3297 |
| 5 | Limit to Year > 2016 | 1678 |

# PsycINFO

| **APA PsychInfo 1806 to June 2025 Week 3**  **Accessed via ovid.com \| Run Date: 25^th^ June 2025** | | |
| --- | --- | --- |
| **Line#** | **Search String** | **No.** |
| 1  Population: Midwife | midwifery/ or (“maternal-child nursing” or “obstetric nursing” or “nurse midwi*e*” or “birth assistant*” or “birth attendant*” or (midwi*e* adj3 (staff or workforce or personnel or student or undergraduate))).ti,ab,id. | 2595 |
| 2  Concept: Recruitment and Retention | human resource management/ or employee retention/ or employee turnover/ or personnel recruitment/ or personnel selection/ or employment policy/ or job applicants/ or employee engagement/ or employment/ or occupational mobility/ or return to work/ or reemployment/ or unemployment/ or occupational choice/ or employee absenteeism/ or employee leave benefits/ or workforce/ or retention/ or (“personnel loyalty” or presenteeism or hiring or hire or staffing or "staff shortages" or attrition or “employee longevity” or ((staff or workforce) adj3 sustainability) or (workforce adj3 stability)).ti,ab,id. or ("intention to leave" or "intention to stay").mp. | 94668 |
| 3 | 1 AND 2 | 73 |
| 4 | Limit to English Language | 73 |
| 5 | Limit to Year > 2016 | 43 |

# Scopus

| **Scopus**  **Accessed via scopus.com \| Run Date: 25^th^ June 2025** | | |
| --- | --- | --- |
| **Line#** | **Search String** | **No.** |
| 1  Population: Midwife | TITLE-ABS-KEY (midwifery OR “midwife” OR “midwives” OR "maternal-child nursing" OR "obstetric nursing" OR "nurse midwives" OR "birth assistant" OR "birth attendant" OR “midwifery staff” OR “midwifery workforce” OR “midwifery personnel” OR “midwifery student” OR “student midwife” OR “student midwives” OR “undergraduate midwife” OR “midwifery undergraduate”) |  |
| 2  Concept: Recruitment and Retention | TITLE-ABS-KEY (“personnel management” OR “job application” OR “personnel loyalty” OR “personnel selection” OR “personnel turnover” OR “work engagement” OR employment OR “career mobility” OR “return to work” OR unemployment OR “career choice” OR “absenteeism” OR “presenteeism” OR “sick leave” OR “workforce” OR hiring OR hire OR recruitment OR “employment policy” OR staffing OR "staff shortages" OR attrition OR “employee longevity” OR retention OR “staff sustainability” OR “workforce sustainability” OR “sustainable workforce” OR “workforce stability” OR "intention to leave" OR "intention to stay”) |  |
| 3 | 1 AND 2 | 5040 |
| 4 | Limit to English Language | 4844 |
| 5 | Limit to Year > 2016 | 2628 |

# CINAHL

| **CINAHL (Cumulative Index to Nursing and Allied Health Literature)**  **Accessed via EBSCOHost.com \| Run Date: 25^th^ June 2025** | | |
| --- | --- | --- |
| **Line#** | **Search String** | **No.** |
| 1  Population: Midwife | XB (midwifery OR “midwife” OR “midwives” OR "maternal-child nursing" OR "obstetric nursing" OR "nurse midwives" OR "birth assistant" OR "birth attendant" OR “midwifery staff” OR “midwifery workforce” OR “midwifery personnel” OR “midwifery student” OR “student midwife” OR “student midwives” OR “undergraduate midwife” OR “midwifery undergraduate”) |  |
| 2  Concept: Recruitment and Retention | XB (“personnel management” OR “job application” OR “personnel loyalty” OR “personnel selection” OR “employment policy” OR “personnel turnover” OR “work engagement” OR employment OR “career mobility” OR “return to work” OR unemployment OR “career choice” OR “absenteeism” OR “presenteeism” OR “sick leave” OR “workforce” OR hiring OR hire OR recruitment OR staffing OR "staff shortages" OR attrition OR “employee longevity” OR retention OR “staff sustainability” OR “workforce sustainability” OR “sustainable workforce” OR “workforce stability” OR "intention to leave" OR "intention to stay”) |  |
| 3 | 1 AND 2 | 2295 |
| 4 | Limit to English Language | 2258 |
| 5 | Limit to Year > 2016 | 1350 |
